# Politics in New Zealand

**Published:** 1905-09

**Authors:** 


### "Politics in New Zealand."

THIS is the title of a pamphlet, one of the quarterly "Equity series," that deals with the politics of a region little understood by the majority of people. It is not a complete work in any sense; it is however, a very satisfying compilation of the subject, taken from "The Story of New Zealand," a pretentious work by Frederick Parsons, selected and arranged by Dr. Taylor who says in an explanatory note:

My purpose has been to place the enlightening and inspiring facts of New Zealand's government and institutions before the people of our country. "The Story of New Zealand," was prepared with great labor and published at great expense, with that purpose in view. * * * It is with a view of placing these political facts within the easy reach of the masses of our people, that I have selected the most important of these facts from the large book and arranged them in this unpretentious pamphlet. I hope that this little book will lead the reader to further studies along the line of progressive government, and particularly do I hope that our people may be inspired to emulate the example of New Zealand, and bring our government as close to the people as that of New Zealand is, and make it serve the interests of the common people.

The main facts of New Zealand's political supremacy, if one may call it such, are set forth in anything but prosy manner, and the story which is in narrative form is illustrated with maps and photoengravings of great interest. It is of course, wholly impossible to set forth any extended review of the book here; but casually, one cannot help being struck forcibly with the socialistic tone of the work in its comparisons between the Utopian luxuries of the fields of labor and politics in New Zealand and the helplessness of the methods in vogue in the United States. Much of that which is said is true; but it is hardly fair to the United States to show by comparative figures that while we have $150,000,000 in warships New Zealand has the same amount in public works, public investment, railways and loans to farmers. The deadly parallel is used to show that conditions in New Zealand are much more beneficent than they are in our own country; that prosperity is more widespread. Did the New Zealand method obtain in the United States we might be more pleased with ourselves individually and as a mass of people; but there would be little use for the "Big Stick" or for some of those other strenuous national attributes which have made us what we are—the most independent, most progressive, most powerful and the richest nation on earth.

Nevertheless, politics in New Zealand is interesting and worth studying.

